# Neuropsychiatric phenotype of post COVID-19 syndrome in non-hospitalized patients

**DOI:** 10.3389/fneur.2022.988359

**Published:** 2022-09-27

**Authors:** Julia Lier, Kristin Stoll, Hellmuth Obrig, Paul Baum, Lea Deterding, Nora Bernsdorff, Franz Hermsdorf, Ines Kunis, Andrea Bräsecke, Sabine Herzig, Matthias L. Schroeter, Angelika Thöne-Otto, Steffi G. Riedel-Heller, Ulrich Laufs, Hubert Wirtz, Joseph Classen, Dorothee Saur

**Affiliations:** ^1^Department of Neurology, University of Leipzig Medical Center, Leipzig, Germany; ^2^Max-Planck-Institute of Human Cognitive and Brain Sciences & Clinic for Cognitive Neurology, University of Leipzig Medical Center, Leipzig, Germany; ^3^Department for Cardiology, University of Leipzig Medical Center, Leipzig, Germany; ^4^Department of Pneumology, University of Leipzig Medical Center, Leipzig, Germany; ^5^Institute of Social Medicine, Occupational Health and Public Health, University of Leipzig Medical Center, Leipzig, Germany

**Keywords:** COVID-19, post COVID-19 syndrome, MFI-20, PCFS, neuropsychiatric disorders

## Abstract

The post COVID-19 syndrome (PCS) is an emerging phenomenon worldwide with enormous socioeconomic impact. While many patients describe neuropsychiatric deficits, the symptoms are yet to be assessed and defined systematically. In this prospective cohort study, we report on the results of a neuropsychiatric consultation implemented in May 2021. A cohort of 105 consecutive patients with merely mild acute course of disease was identified by its high symptom load 6 months post infection using a standardized neurocognitive and psychiatric-psychosomatic assessment. In this cohort, we found a strong correlation between higher scores in questionnaires for fatigue (MFI-20), somatization (PHQ15) and depression (PHQ9) and worse functional outcome as measured by the post COVID functional scale (PCFS). In contrast, neurocognitive scales correlated with age, but not with PCFS. Standard laboratory and cardiopulmonary biomarkers did not differ between the group of patients with predominant neuropsychiatric symptoms and a control group of neuropsychiatrically unaffected PCS patients. Our study delineates a phenotype of PCS dominated by symptoms of fatigue, somatisation and depression. The strong association of psychiatric and psychosomatic symptoms with the PCFS warrants a systematic evaluation of psychosocial side effects of the pandemic itself and psychiatric comorbidities on the long-term outcome of patients with SARS-CoV-2 infection.

## Introduction

According to the British guidelines, the post COVID-19 syndrome (PCS) is defined as a constellation of symptoms which develops following a severe acute respiratory syndrome coronavirus 2 (SARS-CoV-2) infection and persists for more than 12 weeks, while not being explained by an alternative diagnosis (1). Neurological symptoms affecting patients during the acute course of COVID-19 are common and diverse including neuromuscular, cerebrovascular or inflammatory manifestations (2). In contrast, chronic neurological sequelae are less well defined (3). In the UK, a study analyzing retrospective data from over 200.000 patients reported that 12.8% with COVID-19 received a new neurological or psychiatric diagnosis during the first 6 months after initial infection (4). In hospitalized patients, post COVID-19 sequelae were detected in 80%, with a higher risk associated with treatment in the intensive care unit [ICU, (5–7)]. This observation appears to suggest a relationship between the severity of the COVID-19 manifestation and subsequent neuropsychiatric symptom load. However, even young patients who were not hospitalized for COVID-19 and asymptomatic individuals frequently describe neurological and psychiatric sequelae such as anosmia, fatigue, impaired concentration or memory problems months after the infection (8–10). In a meta-analysis covering 39 studies investigating acute and chronic symptoms following an infection with SARS-CoV-2, fatigue presented as the most common symptom in patients with PCS (44%), while anosmia was reported by 10% of the patients (11).

Since the neurobiological substrates underlying the neuropsychiatric manifestations of PCS are largely unknown, an accurate description of the clinical presentation is essential to better understand this syndrome. While many studies describe the symptoms reported by the patients, a systematic and objective characterization of the neuropsychiatric PCS phenotype is still pending. In this prospective study, we present a cohort of 105 consecutive patients from our neurological post COVID-19 consultation examined by a standardized neuropsychiatric assessment. Our main aim was to better understand which neurological, cognitive, psychiatric and psychosomatic symptoms mostly affect the functional long-term outcome of patients with SARS-CoV2 infection. In addition, a control cohort allowed us to compare clinical data, as well as laboratory and cardiopulmonary biomarker profiles between patients with and without neuropsychiatric symptoms.

## Methods

**Figure 1:**
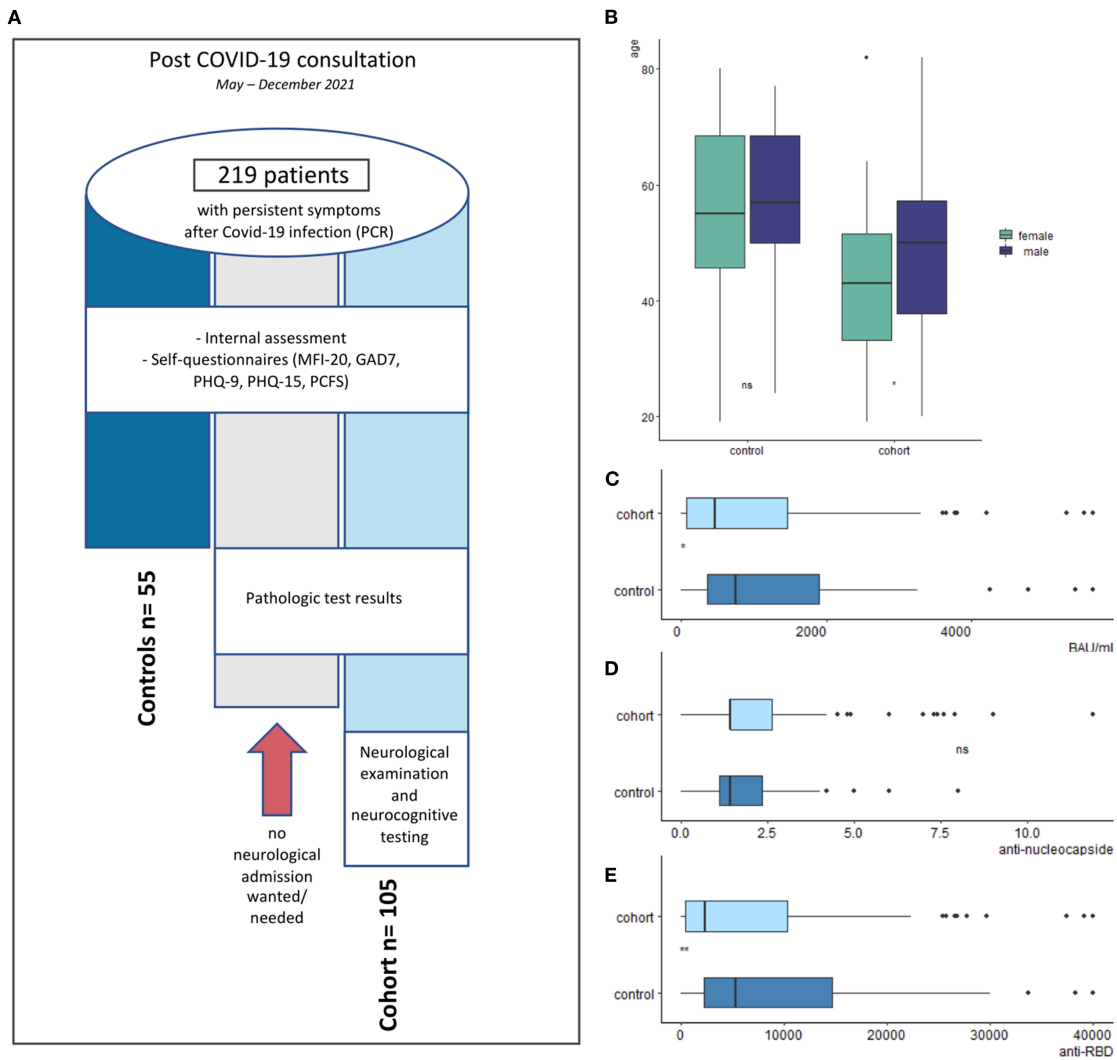
Study design and description of study cohort. **(A)** Flowchart of patient distribution. **(B)** Age between cohort and control. **(C–E)** Comparison of antibody levels between cohort and control. **(E)** Concentration of neutralizing antibodies (binding antibody units per milliliter (BAU/ml) tended to be higher in the control (*p* = 0.012). While the concentration of anti–nucleocapside antibodies did not differ, the control group had significantly higher concentrations of antibodies against the receptor binding domain (anti–RBD; *p* = 0.0046), however possible vaccination–associated influences were not examined.

In May 2021, we implemented an interdisciplinary outpatient clinic for patients suffering from health complaints after a documented infection with SARS-CoV-2, proven by PCR testing. These patients were referred by their general practitioner and primarily seen by an internal medicine specialist. During the initial contact, a thorough cardiopulmonary assessment, standard cardiopulmonary biomarkers (Table 1), SARS-CoV-2 PCR testing on nasopharyngeal swab samples, IgG antibody testing against the spike protein (receptor binding domain, RBD) and nucleocapsid (NC) to confirm the immunological response to the SARS-CoV-2 infection, and the Post COVID Functional Scale (PCFS) were performed. Additionally, several self-questionnaires, including the Multidimensional Fatigue Inventory (MFI-20), Patient Health Questionnaires 9 and 15 (PHQ-9, PHQ-15), the Generalized Anxiety Disorder scale 7 (GAD-7) were used as a basic psychiatric-psychosomatic assessment. When scores in the self-questionnaires were above predefined cut-offs (see below) or the patients reported neuropsychiatric symptoms, a neurological consultation was offered to the patients, if the symptoms were not explained by an alternative diagnosis. In order to further assess the reported deficits possibly associated with PCS, a full neurological examination and neurocognitive testing was performed (Figure 1A). The neurocognitive tests were conducted by a trained medical assistant (IK). All individuals gave their written consent for the scientific use of their data.

**Table 1:** Descriptive statistics.

	**Range**	**Total cohort**	**Controls**	**Study cohort**	***p*–value**
*n*		219	55	105	
Female (*n*, %)		142; 64.5	28; 50.9	69; 66	
Age (median, IQR;[years])		49; 36.75–58.25	56; 48–68.5	44.5; 34–55.75	<0.001
Time post infection [months]		7; 5–9	9;6–10	6; 4–8	<0.001
BMI (median, IQR)		26.1; 23.1–30.2, NA 1	27.6; 24.1–29.7; NA 1	25.6; 22.8–30.7	0.52
**Psychiatric premorbidities**				
Total (*n*, %)		30; 13.6	4; 7.3	16; 15.1	
Depression *(n)*		22	3	14	
Anxiety *(n)*		5	1	1	
PTSD *(n)*		3	0	1	
**Cardiopulmonary biomarkers**				
RR syst (median, IQR; [mmHg])		140; 129.8–155; NA 4	145; 130.2–157.5; NA 1	140; 128–151; NA 3	0.06
RR dist (median, IQR; [mmHg])		85; 78–93; NA 4	82.5; 79.3–91.5; NA 1	85; 78.5–94.5; NA 3	0.74
LVEF (median, IQR; [%])		62; 58–66; NA 41	63; 59–65; NA 6	62; 59–66; NA 30	0.87
FEV1 (median, IQR; [%])		97.1; 89.85–105.6; NA 101	95.8; 90.2–109.25; NA 36	96.5; 91–105.35; NA 39	0.68
**Laboratory biomarkers**				
HbA1c (median, IQR; [%])		5.5; 5.2–5.7; NA 2	5.6; 5.3–5.8;NA 1	5.4; 5.2–5.6; NA 1	0.006
GFR (CKDEPI; median, IQR; [ml/min/1.73 m2)		86; 74–98.25	78; 69–90	87.5; 75–100	0.006
IL−6 (median, IQR; [pg/ml])		1.75; 1.75–1.75; NA 2	1.75; 1.75–1.75; NA 1	1.75; 1.75–1.75	0.19
CRP (median, IQR; [mg/l])		1.12; 0.62– 2.39; NA 1	1.1; 0.68–1.6; NA 1	1; 0.52–2.48	0.46
Ferritin (median, IQR; [μg/l])		98.45; 40.65– 200; NA 2	124; 49.3–259.8; NA 1	96.9; 40.7–182.5	0.58
**Self–questionnaires**				
MFI−20 (median, IQR)	20–100	63; 50–75.25	42; 29.5–51.5	71; 61–81.75	<0.001
PHQ−9 (median, IQR)	0–27	8; 4–12	3; 1–4.5	10.5;8–14	<0.001
PHQ−15 (median, IQR)	0–30	12; 7–16	5; 3–7	14; 10–18	<0.001
GAD−7 (median, IQR)	0–21	6; 3–9	2; 0–4	7; 5–11	<0.001
**PCFS**	0–4	2;1–2	0;0–1	2;2–3	<0.001
**Immune status**				
Anti–nucleocapside (median, IQR; [S/CO])		1.4; 1.1–2.3; NA 10	1.4; 1.1–2.325; NA 3	1.4; 1.4–2.6; NA 1	0.36
Anti–RBD (median, IQR; [AU/ml])		3763; 571–12502; NA 7	5256; 2148–14666; NA 2	2250; 376.2–10273; NA 1	0.0046
BAU/ml (median, IQR)		1243; 85.9–1759; NA 15	746.4; 356–1895; NA 6	465; 66–1464; NA 4	0.012

RR syst, systolic blood pressure; RR diast, diastolic blood presure; LVEF, left ventricular ejection fraction, FEV1, forced exspiratory volume; GFR (CKDEPI), Glomerular filtration rate (Chronic Kidney Disease Epidemiology Collaboration); MFI−20, Multidimensional Fatigue Inventory; PHQ9 and 15, Patient Health Questionnaire 9 (depression module) and 15 (somatisation module); GAD−7, Generalized anxiety disorder scale; anti–RBD, antibodies against receptor binding domain; BAU/ml, binding antibody units per milliliters; IQR, interquartile range; NA, not available.

### Post COVID functional scale (PCFS)

The five-point PCFS was introduced to monitor the functional long-term effects of COVID-19 (12). Even though it is currently not validated, several groups have found an association between a high PCFS score and treatment in the intensive care unit or need for oxygen supplementation during the acute course of illness (13). In an observational study, 70.5% of the analyzed COVID-19 patients described a fully recovered functional status six month after the acute infection (14). For our study, we translated the PCFS into German (Supplementary Figure 1). The PCFS was applied twice, at the initial contact and again at the neurological consultation by the neurologist. In case of discrepancies, the value of the second PCFS was used as primary functional outcome measure.

### Multidimensional fatigue inventory (MFI-20)

The MFI-20 is a self-questionnaire and consists of five subscales covering different domains of fatigue, i.e., general fatigue, physical fatigue, reduced activity, reduced motivation and mental fatigue. The subscores in each domain range from 4 to 20, with higher scores indicating higher levels of fatigue. The MFI-20 was validated in various clinical and healthy cohorts (15) and has since been widely used to assess the severity of fatigue. Currently, there are no strict cut-off values (16). For descriptive statistics, we included (i) the exact values of the subscores for each patient. (ii) the number of domains, where the result was above the third quartile considering the mean values in the general population (16) and (iii) the total value in the MFI-20.

### Patient health questionnaire-9 (PHQ-9)

The PHQ9 is a short and reliable self-questionnaire, scoring each of the nine DSM-IV criteria for depressive disorders. The score ranges between 0 to 27 with higher values indicating more severe depressive symptoms. Scores from 10 had a sensitivity of 88% and a specificity of 88% for major depression (17), making it a sufficient tool in detecting depressive disorders. Accordingly, in our study, scores from 10 were used as indicator for a clinically relevant depression.

### Patient health questionnaire-15 (PHQ-15)

The PHQ15 self-questionnaire is the somatisation module of the PHQ and consists of 13 questions regarding somatoform disorders and two questions from the depressive disorders module asking about sleep disorders and lack of energy (18). The score ranges between 0 to 30 with higher values indicating a more severe somatisation. Significant correlations of health anxiety with illness behavior were described (19). The questionnaire was validated in different cohorts with scores of 5, 10 and 15 representing cut-off values for low, medium and high somatic symptom severity (18). In our study, scores of 10 or more were considered as an indicator for a relevant somatisation disorder.

### Generalized anxiety disorder scale 7 (GAD-7)

The GAD7 is a self-questionnaire and screening tool for general anxiety disorder (GAD) but also for panic, social anxiety and PTSD. It consists of seven items which describe the most important diagnostic criteria for GAD after the DSM-IV. The score ranges from 0–21 with higher values indicating a more severe disorder. Using a cut-off score of 10, it had a sensitivity value of 0,89 and a specificity value of 0,82 for diagnosing GAD (20). Accordingly, in our study we used a cut-off score of 10 as an indicator for GAD.

### Clinical examination and neurocognitive screening

The clinical examination includes a full neurological status with testing of cranial nerves, motor, sensory and coordination functions. Neurocognitive screening consists of questions to test orientation, memory (number span forward/backward, delayed recall of three words), abstract thinking, language and praxis. The neurocognitive screening was mainly used to obtain a test-independent impression of the cognitive level of the patients.

### Sniffin' sticks 12-identification test (SIT-12)

The SIT-12 is a test of nasal chemosensory performance. It consists of a battery of odorant-filled pens. Due to COVID-19-associated hygiene standards, these pens were used to create a line of two centimeters on a neutral fragrance strip. The patients were then asked to smell 3 cm in front of both nostrils and to identify the correct odorant from a list of four descriptors. The odorants are selected to be applicable to the general European population (21). Validated in several countries, a Danish study detected a mean identification score of 11 out of possible 12 among normosmic healthy adult participants (22). In our study, we used a cut-off value <9 as an indicator for hyposmia.

### Montreal cognitive assessment (MoCA)

The MoCA is a brief cognitive screening tool with high sensitivity and specificity to detect a mild cognitive impairment (23). The score ranges between 0 to 30, with higher values indicating better performance. We used the original cut-off score of <26 as indicator for cognitive impairments. When deficits were detected during testing, elements were repeated during the neurocognitive exploration in order to verify the deficit.

### Trailmaking test (TMT) A and B

The TMT consists of two parts, where the participant is instructed to connect a set of 25 dots as quickly as possible while still maintaining accuracy. In TMT A, the dots depict the numbers 1 to 25 and the participant is supposed to connect the numbers in the right order without lifting the pen from the paper. This version is used to examine cognitive processing speed. In TMT B, the participant is asked to alternate between numbers from 1 to 13 and letters from A to L. This part is used to examine executive functioning (24). The time is stopped with a clock in seconds. In our study, we used a modified version for younger populations and applied cut-off values adapted for age and education (25). A percentile ranking <16 was judged as abnormal.

### Semantic verbal fluency test

The semantic verbal fluency test is a short test of verbal executive functioning. In the standard versions of the test, participants are given 1 min to produce as many unique words as possible within a semantic category. The participant's score in each task is the number of unique correct words within 1 min. In our study, we used the category “animal” and applied age and education adapted cut-off scores as suggested by Aschenbrenner et al. (26). Again, a percentile ranking <16 was judged as abnormal.

### Statistical analyses

Statistical analyses were performed using R (Version 4.1.2, http://www.R-project.org). Parameters were tested for normal distribution using Shapiro-Wilk test. For normally distributed data, parametric tests such as *t*-test and Pearson correlation were used. In case of non-parametric data or extreme outliers, we used non-parametric tests such as Mann-Whitney-U-test or Spearman correlation. To adjust the *p*-value for multiple comparison, *post-hoc* Bonferroni correction was performed if needed. A *p*-value <0.05 was considered significant.

## Results

From May to December 2021, 219 consecutive patients visited our interdisciplinary post COVID outpatient clinic. Of these, 105 individuals (48%, female *n* = 69, 66%) with a median age of 44.5 years were transferred to the neurological consultation based on the scores in the initial self-questionnaires or their complaints. This group formed the principal study cohort. 55 individuals (25%, female *n* = 28, 51%) with a median age of 56 years showing no deficits in the psychiatric-psychosomatic self-questionnaires assessed during the first consultation acted as control cohort for the parameters outside the neuropsychiatric assessment (Table 1). The remaining 59 patients did not want a neurological consultation despite (single) scores in the self-questionnaires were above the predefined cut-offs (Figure 1).

### Cardiopulmonary and laboratory biomarkers

While cardiopulmonary and inflammatory markers such as the left ventricular ejection fraction (LVEF), forced exspiratory volume (FEV1) or C-reactive proteine (CRP) did not differ, renal function and HbA1c differed significantly between both groups, a phenomenon which we attributed to the younger median age of the principal cohort (Table 1, Figure 1). All PCR testings for SARS-CoV-2 were negative at the time of admission. In the total post COVID-19 outpatient cohort, RBD- antibodies were positive in 92.2% and NC-antibodies in 56.2%, demonstrating seropositivity in most patients. Interestingly, the study cohort had significantly lower levels of RBD-antibodies and concentrations of neutralizing antibodies (binding antibody units per milliliters, BAU/ml; Table 1, Figures 1C–E). However, since the levels of NC-antibodies decrease with time after infection, whereas the levels of anti-RBD antibodies increase, a vaccination-related effect on anti-RBD must be considered. This, however, was not examined systematically, since the vaccination status was not documented during the whole study period.

### Functional outcome in the study cohort

94.3% (*n* = 99) of the study cohort were home-isolated with no or mild symptoms during the acute course of infection. The median time of consultation was 6 months post infection (IQR 4–8). Notably, 89% of the patients were younger than 60 (*n* = 93). Two thirds of the patients referred to the neurological consultation were women, who were significantly younger than the men in our cohort (female median age = 43, IQR 34–52, male median age = 49.5, IQR 38–57; *p* = 0.046). However, none of the tests or questionnaires displayed a significant difference between male and female patients (Supplementary Table 1). The median PCFS in our study cohort was 2, reflecting slight to moderate functional limitations in everyday life. The number of pre-COVID morbidities and the number of medications taken by the patient correlated significantly with the PCFS (ρ = 0.28, *p* = 0.003). At the time of consultation, 27.6% of the patients were still out of work due to persisting symptoms after the SARS-CoV-2 infection (Table 2). Furthermore, 60% made use of rehabilitation measures such as neurocognitive training or psychological support or somatic rehabilitation.

**Table 2:** Demographic and clinical data of the study cohort with neurological consultation.

**Comorbidities**		Mean (Max;NA) = 1.65 (6;1)
**Medications**		Mean (Max;NA) = 1.72 (10;2)
**Education [years]**		Median(IQR) = 13;13–16
**Acute COVID−19 features**		
No/mild symptoms		94.3% (*n =* 99)
Hospitalization (non–ICU)		2.86% (*n =* 3)
ICU care		0.95% (*n =* 1)
Unknown		1.9% (*n =* 2)
**Functional outcome**		
Sick leave		27.6% (*n =* 29)
Part–time job		9.5% (*n =* 10)
Full–Time Job		39.05% (*n =* 41)
Unemployed		1.9% (*n =* 2)
Retired		5.7% (*n =* 6)
Unknown		16.2% (*n =* 17)
**Treatment**		
No aftercare		33.3% (*n =* 35)
Neurocognitive training		30.48% (*n =* 32)
Psychosomatic support		25,7% (*n =* 27)
Rehabilitation		9.5% (*n =* 10)
Unknown		6.67% (*n =* 7)

ICU, intensive care unit.

### Clinical neurological examination

The clinical neurological examination was unremarkable in two thirds of the patients. Mild pallhypaesthesia or hearing deficits were detected in the remainder, with no clear links to the SARS-CoV-2 infection (Table 3). One patient suffered from critical illness neuromyopathy as a direct result of the intensive care medicine during the acute course of the disease. Regarding olfaction, <9 correctly identified odors in the SIT-12 were detected by 15.6% (*n* = 17) of the patients, indicating mild to more severe olfactory deficits.

**Table 3:** Neurological examination and neurocognitive testing of the study cohort (*N* = 105).

**Test**		**Median (IQR;NA)**	**Min–Max**	**Cut–off**	**Pathologic tests *n* (%)**
**Neurostatus**				
Non-descript					22 (21)
	Pallhypaesthesia				16 (15.24)
	Critical illness myopathy				1 (0.95)
	Other sensory deficits				5 (4.7)
**SIT−12**	Hyposmia	10 (9–11;4)	3–12	<9	17 (15.6)
**Bed side test**					
	Number sequence forward	6 (5–6;42)	3–8	<5	2 (1.9)
	Number sequence backward	4 (4–5;44)	3–6	<4	10 (9.5)
	Delayed recall	3(2–3;42)	0–3	<3	25 (23.8)
**MoCA**		27 (25–28)	16–30	26	37 (35.2)
	#Visuospatial	4(4–5)	1–5	<5	59 (56.2)
	#Language	5(4–5)	2–5	<4	7 (6.7)
	#Phonemic fluency			<1	56 (53.3)
	#Alertness	6(5–6)	3–6	<5	7 (6.7)
	#Abstraction	2(2–2)	0–2	<2	21 (20)
	#Memory	4(3–5)	0–5	<4	48 (45.7)
	#Orientation	6(6–6)	5–6	<5	0 (0)
**Semantic fluency [words]**		24.5 (20–29;3)	10–43	age– and education adapted	15 (14.3)
**Trailmaking**				
	TMT A [seconds]	31 (23–39.75;3)	14–89	age– and education adapted	32 (30.5)
	TMT B [seconds]	54 (44–74;4)	22–160	age– and education adapted	29 (27.2)
**MFI−20**		72 (61–82)	40–97	no validated cut–off	
	#1 General fatigue	16 (14–19; 4)	9–20	no validated cut–off	
	#2 Physical fatigue	16 (13–17; 4)	5–20	no validated cut–off	
	#3 Reduced activity	15 (12–17; 4)	6–20	no validated cut–off	
	#4 Reduced motivation	14 (12–17; 4)	6–20	no validated cut–off	
	#5 Mental fatigue	11 (8–14; 4)	4–19	no validated cut–off	

SIT−12, Sniffin' Sticks−12 identification test; MoCA, Montreal cognitive assessment; in the subtest of phonemic fluency, a number of words <11 was used as cut–off. MFI−20, Multidimensional Fatigue Inventory; IQR, interquartile range; NA, not available.

### Psychiatric-psychosomatic self-questionnaires

**Figure 2:**
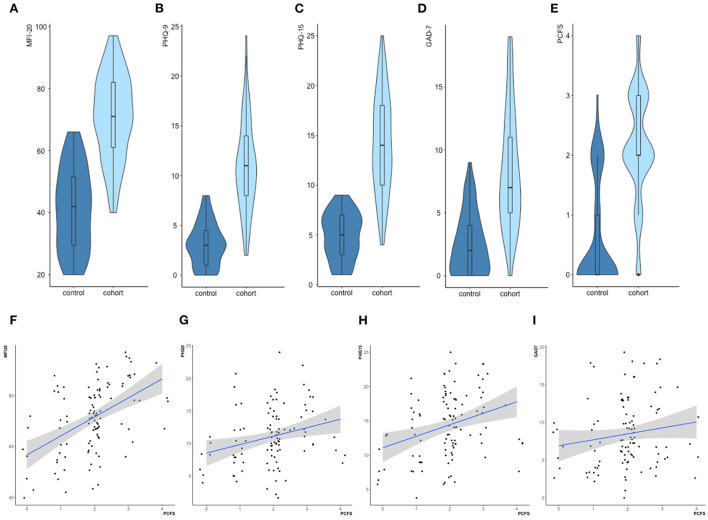
Psychiatric–psychosomatic assessment. **(A–E)** The study cohort revealed significantly higher test results in all psychiatric–psychosomatic self–questionnaires when compared to the neuropsychiatrically unaffected control cohort (all *p*-values < 0.001). **(F–I)** Significant correlations of the Post COVID Functional Scale (PCFS) with the total scores of the MFI–20 (**F**ρ = 0.66, *p* < 0.001), PHQ–9 (**G**ρ = 0.59, *p* < 0.001) and PHQ–15 (**H**ρ = 0.56, *p* < 0.001) and GAD–7 (**I**ρ = 0.4, p < 0.001) in the total cohort (N = 219).

As prespecified by our experimental design, the study cohort revealed significantly higher scores in all psychiatric-psychosomatic self-questionnaires compared to the control cohort (Table 1, Figures 2A–E). A persistent exhaustion since the infection was the most often reported symptom. Eighty four patients (80%) of our study cohort described symptoms in at least four domains of fatigue tested in the MFI-20. Furthermore, there was a strong significant correlation of the overall results in the MFI-20 with the PCFS (ρ = 0.66, *p* < 0.001; Figure 2F). In contrast to the existing literature (16), there was no association of fatigue with age or a specific gender (Supplementary Figure 2). A positive correlation with the PCFS was also seen for the scores in the somatisation module PHQ-9 (Figure 2G), the depression module PHQ-15 (Figure 2H) and the anxiety module GAD-7 (Figure 2I). Analyzing the subgroup who did not receive hospitalization (*n* = 99) did not change these results (Supplementary Table 1).

### Neurocognitive testing

**Figure 3:**
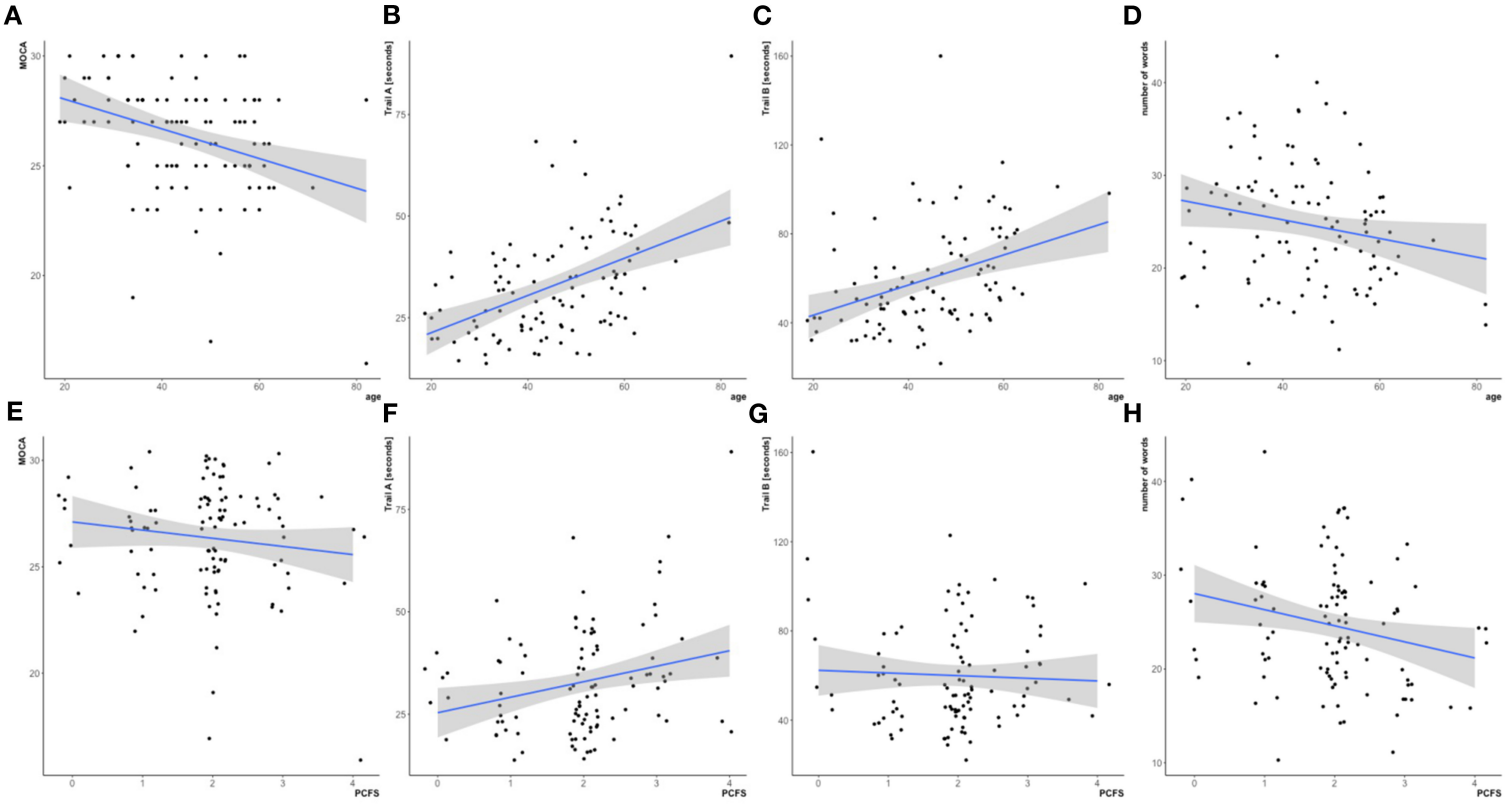
Neurocognitive assessment in the study cohort (*N* = 105). Upper row, correlation with age: Significant correlations of the MoCA (**A** ρ = −0.34, *p* < 0.05), time [in seconds] in the Trail making test A (**B** ρ = 0.44, *p* < 0.05) and Trail making test B (**C** ρ = 0.44, *p* < 0.05) with age. Correlation of number of correct words in the semantic fluency task with age did not stay significant after adjustment for multiple comparisons (**D** ρ = −0.21, *p* = 0.34). Lower row, correlation with post COVID functional scale (PCFS): Not significant correlations of the MoCA (**E** ρ = −0.06, *p* = 1), time [in seconds] in the Trail making A (**F** ρ = 0.2, *p* = 0.33) and Trail making B (**G** ρ = 0.08, *p* = 1) and the number of correct words in the semantic fluency task (**H**, ρ = −0.2, *p* = 0.2) with the PCFS.

35.2% of the patients of our study cohort showed slight impairments in the MoCA when applying our predefined cut-off value (Table 3). Deficits were detected for memory, letter fluency and visuospatial functions. However, we frequently noted that similar tasks could often be performed flawlessly and with greater ease during the clinical neurocognitive exploration. While 56 patients failed the letter fluency test in the MoCA, only 11 of them showed relevant deficits in the additional semantic verbal fluency test. Furthermore, 17 patients failed the MoCA memory task, while demonstrating an error-free delayed recall on clinical examination. Errors in orientation, abstraction, alertness and language were rarely relevant (Table 3). While results in the neurocognitive testing correlated with age (Figures 3A–D), they did not correlate with the PCFS (Figures 3E–H).

## Discussion

In this study, we describe the neuropsychiatric phenotype of the PCS in a prospective cohort of patients 6 months after an acute SARS-CoV-2 infection that did not require hospitalization. Despite favorable cardiopulmonary recovery, most patients still suffered from slight to moderate functional limitations in everyday life. Functional outcome highly correlated with the symptoms of fatigue, depression and somatisation, while no correlation was found with the neurocognitive scores.

All patients of our study cohort underwent a systematic neuropsychiatric assessment. Except for hyposmia in about 15% of the patients, the clinical neurological examination remained unremarkable for COVID-19 associated deficits. However, many patients reported difficulties in memory or attention. Neurocognitive testing detected slight neurocognitive impairments in about one third of the patients. However, discrepant results between the neurocognitive testing and the clinical examination were frequent, suggesting some degree of invalidity in our testing (e.g., low sensitivity for cognitive impairments only affecting high-level performance) and/or functional symptom load in the patients. Our results are in line with a recent study of home-isolated patients with neuropsychiatric complaints in which slight cognitive impairments in the MoCA were also found in one third of the patients about 6 months after the infection (27). Using the Mini-Mental State Examination, a large-scale study on multi-organ assessment in non-hospitalized individuals showed no differences compared to a matched control cohort (28). In addition, neuroimaging biomarkers for vascular brain damage and atrophy in that study did not differ between the groups. In contrast to the prominent complaints, formal neurocognitive testing in our study and others has not clearly revealed severe persistent neurocognitive deficits as part of the PCS. Rather, the mild severity of neurocognitive impairments was contrasted with the observation of severe symptoms of fatigue, depression and somatisation which correlated with functional outcome in the PCFS. This suggests that mainly psychiatric and psychosomatic symptoms influence the long-term outcome after a SARS-CoV-2 infection. However, one needs to emphasize, that especially the PHQ-15 covers multiple physical complaints, which might not be detectable by the internal assessment. Hence, it does not necessarily explain a psychiatric cause for these symptoms.

Regarding the pathogenesis of neuropsychiatric manifestations of COVID-19, several studies point to a potential neurotropism of SARS-CoV-2 (29). The virus enters human cells *via* the angiotensin-converting enzyme 2 receptor which is widely expressed throughout the central nervous system (CNS). However, in autopsy samples with a short post mortem interval, SARS-CoV-2 was only detected in the olfactory mucosa, but not in the olfactory sensory neurons or the parenchyma of the olfactory bulb (30), suggesting an effective barrier preventing the entry into the CNS. In this regard, analyses of the cerebrospinal fluid (CSF) of patients with COVID-19 and neurological symptoms suggest that direct CNS infection seems to be rare, given that classical signs of intrathecal CSF inflammation are typically missing and SARS-CoV-2 PCR testing usually remains negative (31). Against this background, persistence of the virus in the CNS therefore seems to be an unlikely explanation for the long-term neuropsychiatric symptoms. Alternative hypotheses include a persistent disruption of the blood-cerebrospinal fluid barrier (31), an ongoing immune-mediated inflammation (32–34) or a disrupted microcirculation (35). However, most studies were performed in *ex vivo* experimental settings or in autopsy samples of patients with SARS-CoV-2 infection, making assumptions on the potential long-term effects in the living brain difficult. Considering the absence of elevated inflammatory biomarkers and missing evidence for persistent virus or viral antigens due to the negative SARS-CoV-2 PCR testing in our cohort, a chronic inflammation driven by the virus itself seems unlikely. While we detected differences in RBD-antibody levels between the neuropsychiatric and the control cohort, the significance of this finding remains unclear. This is because RBD-antibody levels are also induced by vaccination. This conclusion was supported by the fact that the levels of IgG-antibodies against the nucleocapsid did not differ between the neuropsychiatric and the control group. Therefore, we did not find evidence for an enhanced or diminished infection-associated immune response in patients with neuropsychiatric symptoms. In line with that, other studies found no difference of antibody levels in individuals with confirmed COVID-19 with and without PCS (32, 36).

In the light of a missing distinct neurobiological substrate of the neuropsychiatric PCS, psychiatric and psychosocial factors need to be considered. Whiteside et al. (37) examined 54 outpatient patients 6 months after the acute SARS-CoV-2 infection. They found that formal cognitive performance correlated with mood and anxiety, but neither with the severity of the acute disease nor with the cognitive complaints, pointing to the importance of psychological distress for cognitive performance. This is also in line with a meta-analysis examining psychiatric symptoms after infections with other coronaviruses (SARS and MERS). Fifteen percent of the recovered patients described sleep disorders, emotional lability, impaired concentration and fatigue. However, it was not possible to distinguish between an actual pathophysiologic response to the virus infection and the general effects of the pandemic (38). Even a remarkable number of patients who, contrary to their belief, had not even had contracted a SARS-CoV-2 infection, suffered from symptoms of PCS (39). This finding suggests that PCS could be attributed to the negative effects of the pandemic itself, i.e., the increased psychosocial burden, social isolation and existential fears. Most of the patients who came to the neuropsychiatric consultation described their concerns about limitations at work, social anxiety and worries about long-term consequences of the infection. For some of them, psychological distress seems to be exacerbated by public and social media coverage of post-COVID symptoms. Interestingly, our principal study cohort was significantly younger than the control group. While we scientifically cannot explain the age difference based on our data, socioeconomic factors as discussed above could be a reason for the higher sensitivity for complaints after a Covid-19 infection. The overrepresentation of women in our cohort is consistent with results found in multiple studies where female sex was associated with an increased risk of developing symptoms of PCS such as fatigue and cognitive impairments (5, 40). That women may have a higher risk of developing PCS may correspond to the fact that women tend to carry a larger share of the burden of the pandemic than men (41). One needs to discuss the relation of the symptoms of PCS to the psychosocial environment and a weakened psychosocial resilience due to pre-existing psychiatric comorbidities or long-term psychological stress factors, such as single parenting, fear of job loss, and financial difficulties which may affect more women than men. In line with a predominantly psychosocial origin of PCS, in our cohort, premorbid depression was more frequent in the study than in the control cohort. Future studies will need to evaluate the role of psychosocial factors in the pathogenesis of PCS more systematically and in more detail.

Irrespective of the underlying cause of PCS, it is evident that the large number of patients who are still unable to return to their work or activity level before the pandemic poses a severe socioeconomic problem. While reliable numbers of post COVID-19 cases recognized as occupational diseases are still lacking, insurance companies report record numbers in requests (42, 43). Therefore, long-term programs are needed to provide support independently of the underlying cause of persisting symptoms after COVID-19. It seems likely that symptom management will be less successful when based solely on biological rather than incorporating psychosocial concepts of illness. Fortunately, first studies show that the reported cognitive deficits may regress over time (44) and are less likely to appear in vaccinated patients (45).

The rapidly increasing case numbers around the world due to the predominance of the omicron variant might be both, a challenge and a chance. While higher case numbers could mean even more patients suffering from long-term symptoms, the social significance of an infection may decrease, as it becomes more common to become infected by SARS-CoV-2.

## Limitations

There are certain limitations to our study, which we would like to address. First, since our control group also suffered from symptoms due to the SARS-CoV-2 infection, we did not test a healthy control group. Therefore, strict conclusions on the influence of the pandemic itself on neuropsychiatric symptoms remain hypothetical. Secondly, our neurocognitive tests did not allow for the detection of subtler cognitive impairments, in particular those only affecting high-level performance in daily life. Therefore, the contribution of slight cognitive impairments to PCS might be underestimated in our study and future studies should put a particular emphasis on the detection of subtle, but still functionally relevant neurocognitive deficits. This consideration must not neglect the discrepancy between the findings in the clinical neurocognitive testing and the psychiatric-psychosomatic assessment. Thirdly, we did not examine biomarkers for neurodegeneration and brain injury in blood or cerebrospinal fluid. However, although we cannot rule out permanent neuronal injury in individual cases, the results of our neurological and neurocognitive examinations do not indicate persistent organic brain dysfunction.

## Conclusions

In this article, we present a prospective cohort of mainly non-hospitalized patients about 6 months after the acute SARS-CoV-2 infection who present with a clinical phenotype dominated by symptoms of depression, somatisation and fatigue. The strong association of the severity of these symptoms with the PCFS underlines the functional importance of these symptoms for long-term outcome after an infection with SARS-CoV-2. Although we did not focus on the mechanisms underlying the neuropsychiatric manifestations of PCS, our findings provide indirect evidence to suggest that PCS is strongly influenced by psychosocial consequences of the pandemic itself and by premorbid psychiatric and psychosomatic comorbidities.

## Data availability statement

The raw data supporting the conclusions of this article will be made available by the authors, without undue reservation.

## Ethics statement

Ethical review and approval was not required for the study on human participants in accordance with the local legislation and institutional requirements. The patients/participants provided their written informed consent to participate in this study.

## Author contributions

JL, FH, NB, SH, HO, AT-O, and MS carried out the neurological consultation. KS assisted in data collection and preparation. IK and AB carried out the cognitive and neuropsychiatric testing. PB, LD, HW, and UL carried out the internal consultation and contributed the laboratory data. SH, HO, AT-O, MS, PB, SR-H, and DS were involved in planning the prospective study procedures. JL processed the experimental data, performed the analysis, drafted the manuscript, and designed the figures. JC contributed to the implementation of the research and revised the manuscript thoroughly. DS supervised the project. All authors provided critical feedback and helped shape the research, analysis, and manuscript.

## Conflict of interest

The authors declare that the research was conducted in the absence of any commercial or financial relationships that could be construed as a potential conflict of interest.

## Publisher's note

All claims expressed in this article are solely those of the authors and do not necessarily represent those of their affiliated organizations, or those of the publisher, the editors and the reviewers. Any product that may be evaluated in this article, or claim that may be made by its manufacturer, is not guaranteed or endorsed by the publisher.

## Supplementary material

Supplementary Table 1 Statistics of cognitive and neuropsychiatric tests.

Supplementary Figure 1 German translation of post COVID functional scale. **(A)** Flowchart. **(B**) Patient questionnaire. Following the instructions given by Klok et al., (12), the PFCS is used to assess recovery after the SARS–CoV−2 infection.

Supplementary Figure 2 Analysis of the MFI subscales in the study cohort (*N* = 105) showed no differences between male and female patients **(A–E)**. An analysis of the MFI−20 total score did not show a significant correlation with age **(F)**, (ρ = 0.17, p.adj. = 0.58).

## References

[1] Shah W Hillman T Playford ED Hishmeh L. Managing the long-term effects of covid-19: summary of NICE, SIGN, and RCGP rapid guideline. BMJ. (2021) 372:n136. 10.1136/bmj.n136 33483331

[2] Pezzini A Padovani A. Lifting the mask on neurological manifestations of COVID-19. Nat Rev Neurol. (2020) 16:636–44. 10.1038/s41582-020-0398-3 32839585 PMC7444680

[3] Balcom EF Nath A Power C. Acute and chronic neurological disorders in COVID-19: potential mechanisms of disease. Brain. (2021) 144:3576–88. 10.1093/brain/awab302 34398188 PMC8719840

[4] Taquet M Geddes JR Husain M Luciano S Harrison PJ. 6-month neurological and psychiatric outcomes in 236 379 survivors of COVID-19: a retrospective cohort study using electronic health records. The Lancet Psychiatry. (2021) 8:416–27. 10.1016/S2215-0366(21)00084-5 33836148 PMC8023694

[5] Halpin SJ McIvor C Whyatt G Adams A Harvey O McLean L et al. Postdischarge symptoms and rehabilitation needs in survivors of COVID-19 infection: A cross-sectional evaluation. J Med Virol. (2021) 93:1013–22. 10.1002/jmv.26368 32729939

[6] Huang C Huang L Wang Y Li X Ren L Gu X et al. 6-month consequences of COVID-19 in patients discharged from hospital: a cohort study. Lancet. (2021) 397:220–32. 10.1016/S0140-6736(20)32656-8 33428867 PMC7833295

[7] Raman B Cassar MP Tunnicliffe EM Filippini N Griffanti L Alfaro-Almagro F et al. Medium-term effects of SARS-CoV-2 infection on multiple vital organs, exercise capacity, cognition, quality of life and mental health, post-hospital discharge. EClinicalMedicine. (2021) 31:100683. 10.1016/j.eclinm.2020.100683 33490928 PMC7808914

[8] Blomberg B Mohn KG-I Brokstad KA Zhou F Linchausen DW Hansen B-A et al. Long COVID in a prospective cohort of home-isolated patients. Nat Med. (2021) 27:1607–13. 10.1038/s41591-021-01433-3 34163090 PMC8440190

[9] Graham EL Clark JR Orban ZS Lim PH Szymanski AL Taylor C et al. Persistent neurologic symptoms and cognitive dysfunction in non-hospitalized Covid-19 “long haulers”. Ann Clin Transl Neurol. (2021) 8:1073–85. 10.1002/acn3.51350 33755344 PMC8108421

[10] Tenforde MW Kim SS Lindsell CJ Billig Rose E Shapiro NI Files DC et al. Symptom duration and risk factors for delayed return to usual health among outpatients with COVID-19 in a Multistate Health Care Systems Network - United States, March-June 2020. MMWR Morb Mortal Wkly Rep. (2020) 69:993–8. 10.15585/mmwr.mm6930e1 32730238 PMC7392393

[11] Jennings G Monaghan A Xue F Mockler D Romero-Ortuño R. A systematic review of persistent symptoms and residual abnormal functioning following acute COVID-19: ongoing symptomatic phase vs. Post-COVID-19 syndrome. J Clin Med. (2021)10:45913. 10.3390/jcm10245913 34945213 PMC8708187

[12] Klok FA Boon GJAM Barco S Endres M Geelhoed JJM Knauss S. The Post-COVID-19 Functional Status scale: a tool to measure functional status over time after COVID-19. Eur Respir J. (2020) 56:2020. 10.1183/13993003.01494-2020 32398306 PMC7236834

[13] Mohamed Hussein AA Saad M Zayan HE Abdelsayed M Moustafa M Ezzat AR et al. Post-COVID-19 functional status: relation to age, smoking, hospitalization, and previous comorbidities. Ann Thorac Med. (2021) 16:260–5. 10.4103/atm.atm_606_20 34484441 PMC8388571

[14] Du H-W Fang S-F Wu S-R Chen X-L Chen J-N Zhang Y-X et al. Six-month follow-up of functional status in discharged patients with coronavirus disease 2019. BMC Infect Dis. (2021) 21:1271. 10.1186/s12879-021-06970-3 34930161 PMC8686090

[15] Smets EMA Garssen B Bonke B De Haes JCJM. The Multidimensional fatigue Inventory (MFI) - psychometric qualities of an instrument to assess fatigue. J Psychosom Res. (1995) 39:315–25. 10.1016/0022-3999(94)00125-O 7636775

[16] Schwarz R Krauss O Hinz A. Fatigue in the general population. Onkologie. (2003) 26:140–4. 10.1159/000069834 12771522

[17] Kroenke K Spitzer RL Williams JBW. The PHQ-9. Validity of a brief depression severity measure. J General Int Med. (2001) (16):606–13. 10.1046/j.1525-1497.2001.016009606.x 11556941 PMC1495268

[18] Kroenke K Spitzer RL Williams JBW. The PHQ-15: validity of a new measure for evaluating the severity of somatic symptoms. Psychosom Med. (2002) (64):258–66. 10.1097/00006842-200203000-00008 11914441

[19] Zijlema WL Stolk RP Löwe B Rief W White PD Rosmalen JGM. How to assess common somatic symptoms in large-scale studies: a systematic review of questionnaires. J Psychosom Res. (2013) 74:459–68. 10.1016/j.jpsychores.2013.03.093 23731742

[20] Spitzer RL Kroenke K Williams JBW Löwe B. A brief measure for assessing generalized anxiety disorder: the GAD7. Arch Intern Med. (2006) 166:1092–7. 10.1001/archinte.166.10.1092 16717171

[21] Kobal G Hummel T Sekinger B Barz S Roscher S Wolf S. “Sniffin' sticks”: screening of olfactory performance. Rhinology. (1996) 34:222–6. 10.1037/t58174-000 9050101

[22] Fjaeldstad A Kjaergaard T van Hartevelt TJ Moeller A Kringelbach ML Ovesen T. Olfactory screening: validation of Sniffin' Sticks in Denmark. Clin Otolaryngol. (2015) 40:545–50. 10.1111/coa.12405 25721152

[23] Nasreddine ZS Phillips NA Bédirian V Charbonneau S Whitehead V Collin I et al. The Montreal Cognitive Assessment, MoCA: a brief screening tool for mild cognitive impairment. J Am Geriatr Soc. (2005) 53:695–9. 10.1111/j.1532-5415.2005.53221.x 15817019

[24] Kopp B Rösser N Tabeling S Stürenburg HJ Haan B de Karnath H-O et al. Errors on the trail making test are associated with right hemispheric frontal lobe damage in stroke patients. Behav Neurol. (2015) 2015:309235. 10.1155/2015/309235 26074673 PMC4444530

[25] Rodewald K Bartolovic M Debelak R Aschenbrenner S Weisbrod M Roesch-Ely D. A normative study of a modified trail making test in a German speaking population. Zeitschrift für Neuropsychologie. (2012) 23:60. 10.1024/1016-264X/a000060

[26] Aschenbrenner S Tucha O Lange KW. Regensburger Wortflüssigkeits-Test: RWT. Hogrefe, Verlag für Psychologie. (2000).

[27] Dressing A Bormann T Blazhenets G Schroeter N Walter LI Thurow J et al. Neuropsychologic profiles and cerebral glucose metabolism in neurocognitive long COVID syndrome. J Nucl Med. (2022) 63:1058–63. 10.2967/jnumed.121.262677 34649946 PMC9258569

[28] Petersen EL Goßling A Adam G Aepfelbacher M Behrendt C-A Cavus E et al. Multi-organ assessment in mainly non-hospitalized individuals after SARS-CoV-2 infection: the Hamburg City Health Study COVID programme. Eur Heart J. (2022) 43:1124–37. 10.1093/eurheartj/ehab914 34999762 PMC8755397

[29] Zubair AS McAlpine LS Gardin T Farhadian S Kuruvilla DE Spudich S. Neuropathogenesis and neurologic manifestations of the coronaviruses in the age of Coronavirus Disease 2019: a review. JAMA Neurol. (2020) 77:1018–27. 10.1001/jamaneurol.2020.2065 32469387 PMC7484225

[30] Khan M Yoo S-J Clijsters M Backaert W Vanstapel A Speleman K et al. Visualizing in deceased COVID-19 patients how SARS-CoV-2 attacks the respiratory and olfactory mucosae but spares the olfactory bulb. Cell. (2021) 184:5932–49.e15. 10.1016/j.cell.2021.10.027 34798069 PMC8564600

[31] Jarius S Pache F Körtvelyessy P Jelčić I Stettner M Franciotta D et al. Cerebrospinal fluid findings in COVID-19: a multicenter study of 150 lumbar punctures in 127 patients. J Neuroinflammation. (2022) 19:19. 10.1186/s12974-021-02339-0 35057809 PMC8771621

[32] Merad M Blish CA Sallusto F Iwasaki A. The immunology and immunopathology of COVID-19. Science. (2022) 375:1122–7. 10.1126/science.abm8108 35271343 PMC12828912

[33] Ryan FJ Hope CM Masavuli MG Lynn MA Mekonnen ZA Yeow AEL et al. Long-term perturbation of the peripheral immune system months after SARS-CoV-2 infection. BMC Med. (2022) 20:26. 10.1186/s12916-021-02228-6 35027067 PMC8758383

[34] Schwabenland M Salié H Tanevski J Killmer S Lago MS Schlaak AE et al. Deep spatial profiling of human COVID-19 brains reveals neuroinflammation with distinct microanatomical microglia-T-cell interactions. Immunity. (2021) 54:1594–1610.e11. 10.1016/j.immuni.2021.06.002 34174183 PMC8188302

[35] Lee M-H Perl DP Nair G Li W Maric D Murray H et al. Microvascular injury in the brains of patients with Covid-19. N Engl J Med. (2021) 384:481–3. 10.1056/NEJMc2033369 33378608 PMC7787217

[36] Pereira C Harris BHL Di Giovannantonio M Rosadas C Short C-E Quinlan R et al. The association between antibody response to severe acute respiratory syndrome coronavirus 2 infection and post-COVID-19 syndrome in healthcare workers. J Infect Dis. (2021) 223:1671–6. 10.1093/infdis/jiab120 33675366 PMC7989400

[37] Whiteside DM Basso MR Naini SM Porter J Holker E Waldron EJ et al. Outcomes in post-acute sequelae of COVID-19 (PASC) at 6 months post-infection Part 1: cognitive functioning. Clin Neuropsychol. (2022) 36:806–28. 10.1080/13854046.2022.2030412 35130818

[38] Rogers JP Chesney E Oliver D Pollak TA McGuire P Fusar-Poli P et al. Psychiatric and neuropsychiatric presentations associated with severe coronavirus infections: a systematic review and meta-analysis with comparison to the COVID-19 pandemic. Lancet Psychiatr. (2020) 7:611–27. 10.1016/S2215-0366(20)30203-0 32437679 PMC7234781

[39] Matta J Wiernik E Robineau O Carrat F Touvier M Severi G et al. Association of self-reported COVID-19 infection and SARS-CoV-2 serology test results with persistent physical symptoms among french adults during the COVID-19 pandemic. JAMA Intern Med. (2022) 182:19–25. 10.1001/jamainternmed.2021.6454 34747982 PMC8576624

[40] Ceban F Ling S Lui LMW Lee Y Gill H Teopiz KM et al. Fatigue and cognitive impairment in Post-COVID-19 syndrome: a systematic review and meta-analysis. Brain Behav Immun. (2022) 101:93–135. 10.1016/j.bbi.2021.12.020 34973396 PMC8715665

[41] O'Donnell M Bourgault S McDougal L Dehingia N Cheung WW Raj A. The Impacts of COVID-19 on Women's Social Economic Outcomes: An Updated Review of the Evidence. CGD Policy Paper 225. (2021). Available online at: https://cnxus.org/wp-content/uploads/2021/10/impacts-covid-19-womens-social-and-economic-outcomes-updated-review-evidence.pdf (accessed September 16, 2022).

[42] Deutsche, Gesetzliche Unfallversicherung e,.V. (DGUV), Glinkastraße 40, 10117 Berlin. Available online at: https://www.dguv.de/medien/inhalt/mediencenter/hintergrund/covid/dguv_zahlen_covid.pdf (accessed September 16, 2022).

[43] Berufsgenossenschaft, für Gesundheitsdienst und Wohlfahrtspflege (BGW),. Pappelallee 33/35/37, 22089 Hamburg. Available online at: https://www.bgw-online.de/bgw-online-de/presse/corona-berufskrankheit-unterstuetzung-post-covid-betroffene-64146 (accessed September 16, 2022).

[44] Del Brutto OH Rumbea DA Recalde BY Mera RM. Cognitive sequelae of long COVID may not be permanent: a prospective study. Eur J Neurol. (2021). 10.1111/ene.15215 34918425

[45] Kuodi P Gorelik Y Zayyad H Wertheim O Wiegler KB Jabal KA. Association between BNT162b2 vaccination and reported incidence of post-COVID-19 symptoms: Cross-sectional study 2020–21, Israel. NPJ Vaccines. (2022) 7:101. 10.1038/s41541-022-00526-5 36028498 PMC9411827

