# Supplementary material for: Neuropsychiatric phenotype of post COVID-19 syndrome in non-hospitalized patients
Source: Front Neurol. 2022 Sep 27;13:988359. doi: 10.3389/fneur.2022.988359 (PMC9552839; doi:10.3389/fneur.2022.988359)
Supplement: Supplementary Table 1 — Statistics of cognitive and neuropsychiatric tests. [file Data_Sheet_1.PDF]

	all patients (n=105)				home-isolated patients (n=99)			
	**method**	**cor**	**p**	**p.adj.**	**method**	**cor**	**p**	**p.adj.**
PHQ15~sex	t.test		0.3	1	t.test		0.35	1
PHQ15~age	pearson	0.13	0.2	1	pearson	0.14	0.17	1
**PHQ15~PCFS**	**spearman**	**0.33**	**<0.001**	**0.0054**	**spearman**	**0.36**	**<0.001**	**0.0017**
PHQ9~sex	t.test		0.2	1	t.test		0,19	1
PHQ9~age	pearson	-0.084	0.4	1	pearson	-0.093	0.36	1
**PHQ9~PFCS**	**spearman**	**0.31**	**0.0012**	**0.01**	**spearman**	**0.33**	**<0.001**	**0.0075**
MFI20~sex	t.test		0.58	1	t.test		0.61	1
MFI20~age	pearson	0.15	0.13	1	pearson	0.14	0.16	1
**MFI20~PFCS**	**spearman**	**0.48**	**<0.001**	**0.002**	**spearman**	**0.47**	**<0.001**	**<0.001**
GAD7~sex	wilcox		0.26	1	wilcox		0.098	0.78
GAD7~age	spearman	0.037	0.71	1	spearman	0.01	0.9	1
GAD7~PFCS	spearman	0.16	0.088	0.70	spearman	0.2	0.054	0.43
MoCa~sex	wilcox		0.41	1	wilcox		0.72	1
**MoCa~age**	**spearman**	**-0.34**	**<0.001**	**0.0036**	**spearman**	**-0.32**	**0.001**	**0.01**
MoCa~PFCS	spearman	-0.059	0.55	1	spearman	-0.054	0.6	1
TrailA~sex	wilcox		0.49	1	wilcox		0.7	1
**TrailA~age**	**spearman**	**0.438**	**<0.001**	**<0.001**	**spearman**	**0.42**	**<0.001**	**<0.001**
**TrailA~PCFS**	**spearman**	**0.203**	**0.041**	0.33	**spearman**	**0.228**	**0.026**	0.205
TrailB~sex	wilcox		0.15	1	wilcox		0.29	1
**TrailB~age**	**spearman**	**0.44**	**<0.001**	**<0.001**	**spearman**	**0.44**	**0.012**	0.087
TrailB~PCFS	spearman	0.08	0.44	1	spearman	0.11	0.302	1
verbal fluency~sex	t.test		0.31	1	t.test		0.59	1
**verbal fluency~age**	**pearson**	**-0.202**	**0.042**	0.34	**pearson**	**-0.223**	**0.03**	0.23
**verbal fluency~PCFS**	**spearman**	**-0.22**	**0.025**	0.2	**spearman**	**-0.244**	**0.017**	0.134
