# Supplementary figures and images for: Neuropsychiatric phenotype of post COVID-19 syndrome in non-hospitalized patients
Source: Front Neurol. 2022 Sep 27;13:988359. doi: 10.3389/fneur.2022.988359 (PMC9552839; doi:10.3389/fneur.2022.988359)

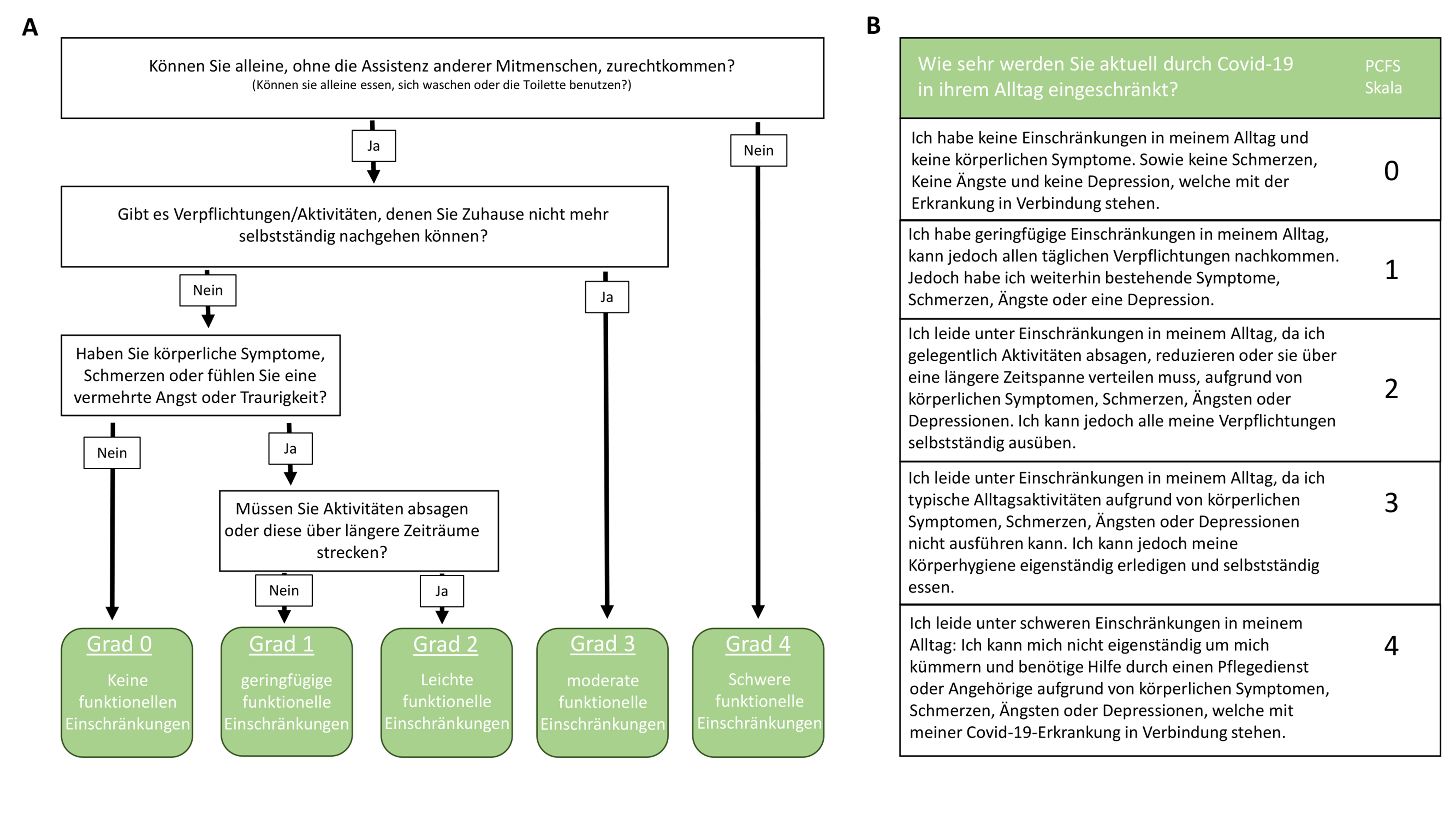

Supplement: Supplementary Figure 1 — German translation of post COVID functional scale. (A) Flowchart. (B) Patient questionnaire. Following the instructions given by Klok et al., (12), the PFCS is used to assess recovery after the SARS–CoV−2 infection. [file Image_1.TIFF]

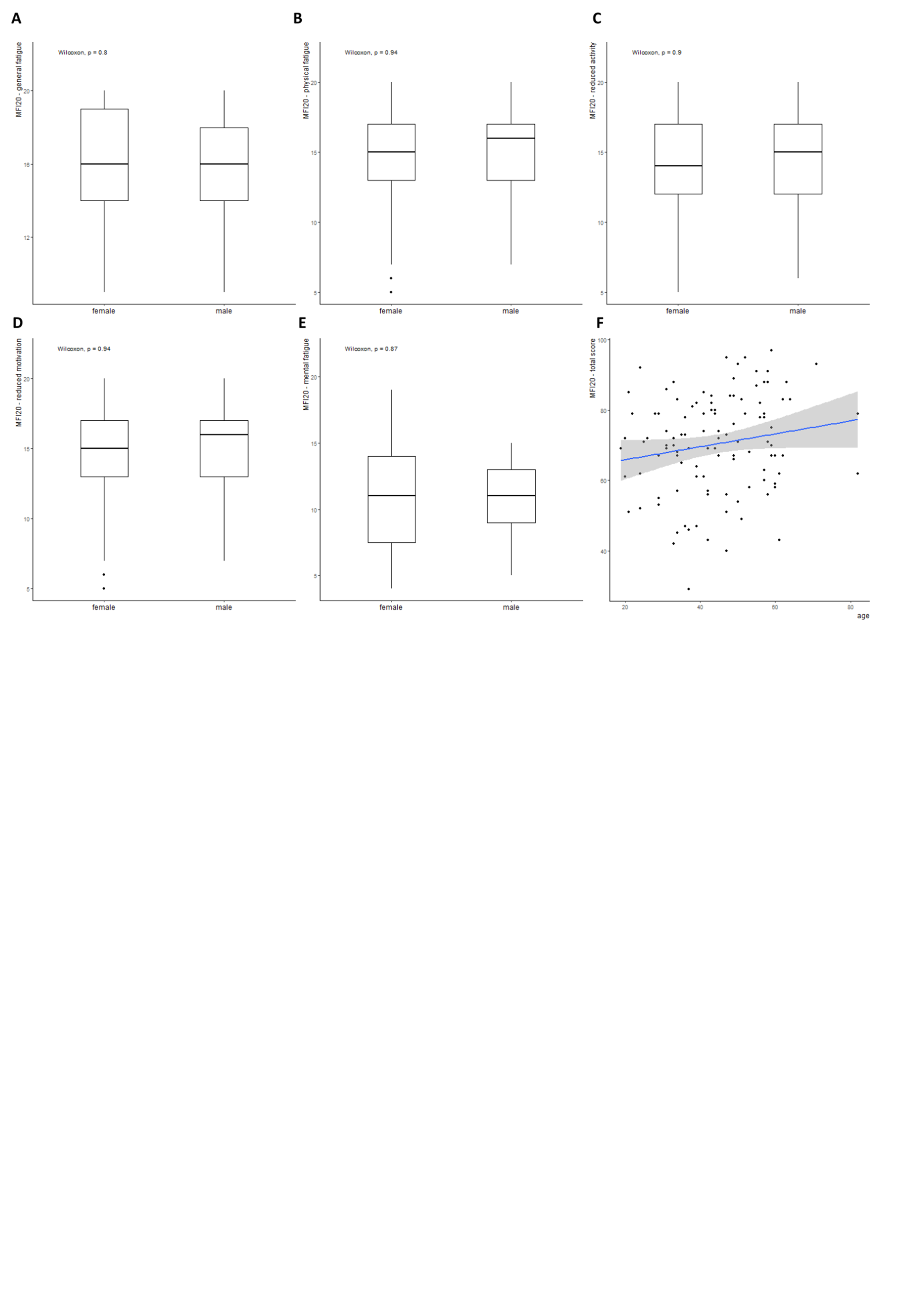

Supplement: Supplementary Figure 2 — Analysis of the MFI subscales in the study cohort (N = 105) showed no differences between male and female patients (A–E). An analysis of the MFI−20 total score did not show a significant correlation with age (F), (ρ = 0.17, p.adj. = 0.58). [file Image_2.TIFF]
